# A Novel Estimation of the Relative Economic Value in Terms of Different Chronic Hepatitis B Treatment Options

**DOI:** 10.1371/journal.pone.0057900

**Published:** 2013-03-11

**Authors:** Jun Yong Park, Jeong Heo, Tae Jin Lee, Hyung Joon Yim, Jong Eun Yeon, Young-Suk Lim, Min Jeong Seo, Sang Hoon Ahn, Myung Seok Lee

**Affiliations:** 1 Department of Internal Medicine, Yonsei University College of Medicine, Seoul, Korea; 2 Department of Internal Medicine, Pusan National University School of Medicine, Busan, Korea; 3 Seoul National University School of Public Health & Institute, Seoul, Korea; 4 Department of Internal Medicine, Korea University College of Medicine, Seoul, Korea; 5 Department of Internal Medicine, University of Ulsan College of Medicine, Seoul, Korea; 6 Monitor Group Korea, Seoul, Korea; 7 Department of Internal Medicine, Hallym University College of Medicine, Seoul, Korea; Mahidol-Oxford Tropical Medicine Research Unit, Thailand

## Abstract

**Background:**

Prescribers, payors and healthcare decision-makers are increasingly examining the value of treatments. This study aims at analyzing economic value of chronic hepatitis B (CHB) treatment options, which are available in Korea.

**Methods:**

CHB infection was simulated using a health-state transition model with disease states defined as mild disease (Ishak F0/F1), fibrosis (F2/F3/F4), advanced fibrosis/cirrhosis (>F4), and complicated disease states (decompensated cirrhosis, hepatocellular carcinoma, liver transplant and death) based on available natural history data. The value of treatment-specific attributes on disease progression/regression was estimated based on published data in terms of events and costs avoided. 5-year treatment duration was assumed except for treatment initiation. Primary model output is the estimated cost savings of entecavir per patient per day of treatment versus the comparator in question for a given CHB patient.

**Results:**

The simulation of treating with entecavir versus no treatment predicted improved clinical outcomes for entecavir-treatment patients. In the long term, these clinical benefits translate into cost savings of $3.10 per day of treatment. In naive patient treatment, daily cost savings of using entecavir versus lamivudine or telbivudine was estimated at $2.89 and $1.72, respectively. In the case of suboptimal responders who pre-treated with lamivudine, daily cost saving for patients switching to entecavir was $1.38 per day of treatment compared to patients maintaining on lamivudine.

**Conclusions:**

Entecavir exhibits characteristics of a favourable CHB treatment, which directly translates into economic and therapeutic value as opposed to either no treatment or alternative strategies.

## Introduction

Chronic hepatitis B (CHB) is an infectious disease caused by hepatitis B virus (HBV). If not successfully treated, CHB can lead to progressive liver damages, including cirrhosis, hepatocellular carcinoma (HCC) and death [1]. Approximately 2 billion people worldwide are infected with HBV and more than 350 million have chronic hepatitis B (CHB) infection (WHO 2010). Although the incidence of HBV infection has been decreasing in Korea, in part because of vaccination strategies and improved socio-economic conditions [2], large number of patients is still affected. In Korea, the incidence of reported hepatitis B cases per 100,000 inhabitants in 2010 was 290 (market report by Synovate 2010). The risk of cirrhosis and HCC is also higher in patients who had persistently high levels of HBV replication (high viral load) and long durations of active hepatitis. These states of the disease are associated with an increased risk of morbidity and mortality, and incur considerable healthcare costs. Treatment for chronic hepatitis B aims to prevent or reduce morbidity and mortality associated with cirrhosis and HCC. It can be achieved with the eradication of HBV infection or clearance of serum HBsAg, but rarely with current antiviral treatment. The more realistic goal is to maintain suppression of HBV replication at the lowest possible level. Several studies have shown that inhibition of viral replication is associated with remission of liver disease and prevention of HBV related complication. Recently, seven therapeutic agents have been approved for treatment of chronic hepatitis B patient. Among these, entecavir, lamivudine, and telbivudine are the most widely used antiviral agents in Asia Pacific region including Korea, where tenofovir is not yet available. Optimal antiviral therapy that induces sustained suppression of HBV replication can modify the natural history of chronic HBV infection [3], thereby reducing the human and financial costs of this disease [4], [5]. The latest CHB treatment guidelines by APASL (Asian Pacific Association for the Study of the Liver Disease) recommend the use of potent antiviral drugs with high genetic barrier. However, these recommendations do not take into consideration treatment cost or therapy monitoring. In fact, the increased financial burden of CHB healthcare requires physicians, payors, and healthcare decision-makers to evaluate the cost-effectiveness of therapy [6], [7]. The benefit of treating CHB patients according to guidelines can be measured by the value of avoiding the costs associated with disease progression.

A perceived value assessment (PVA) model was employed to analyze the long-term impact of CHB treatment scenarios in Korea, aiming to shed light on the relative economic value of each antiviral therapy. The PVA model attempts to capture the long-term complications and effects of treatment through the use of a multilayered Markovian model. The PVA methodology is consistent with a cost-benefit analysis, where the clinical benefits of treatment-occurrence of histological improvement and CHB disease regression, avoidance of resistance, avoidance of renal adverse events, avoidance of additional monitoring requirements-are expressed in monetary terms. The PVA method differs from conventional health economics analysis in that it is designed to disaggregate economic values, each value linked to the aforementioned clinical benefits. The primary output is cost-avoidance against a chosen therapy option, with discrete breakdown by cost-differentiating clinical attribute. We report PVA analysis on available CHB treatment options at treatment initiation, naive patient treatment and suboptimal switch treatment. The results are illustrated as comparison against entecavir, the most potent as well as the most expensive NA antiviral therapy in the market.

## Materials and Methods

### Treatment options

**Figure 1:**
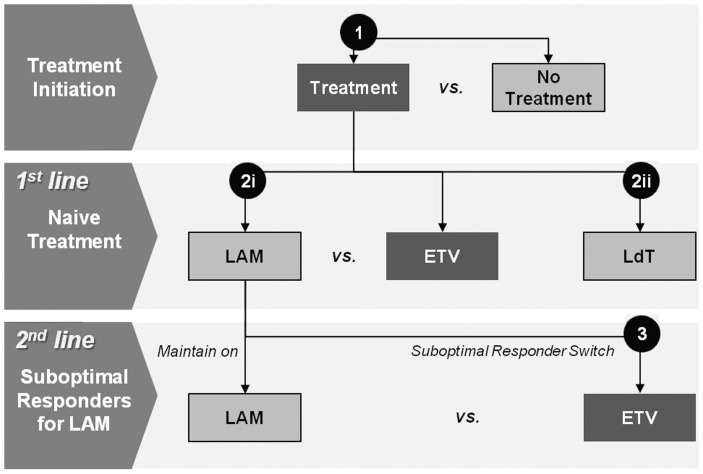
Treatment Options. (1) Treatment option showing comparison between treatment with entecavir versus no treatment, (2i) In naive patient treatment, comparison between treatment with lamivudine versus with entecavir, (2ii) comparison between treatment with telbivudine versus entecavir, (3) In suboptimal responders who pre-treated with lamivudine for a least 1 year without YMDD resistance, comparison between maintained on lamivudine versus switched to entecavir (1 mg).

Long-term clinical and economic evaluation of entecavir was compared against various treatment regimes (Figure 1), including 1) treatment initiation (entecavir treatment vs. no treatment), 2) naive patient treatment, compared with lamivudine (100 mg/day), and telbivudine (600 mg/day), 3) management of suboptimal responders who were pre-treated with, but did not develop resistance to, lamivudine for at least 1 year (switch to entecavir vs. maintain on lamivudine).

### Modelling

To capture the long-term complications and effects of CHB treatment with entecavir, and other available antiviral agents (lamivudine and telbivudine), a model was developed with two distinct but interlinked components. A multilayered Markovian model (disease state transition model) was used to simulate the natural progression of hepatitis B and the effect of treatment. This was combined with a detailed cost calculator for all components in the natural history model and each treatment option.

**Figure 2:**
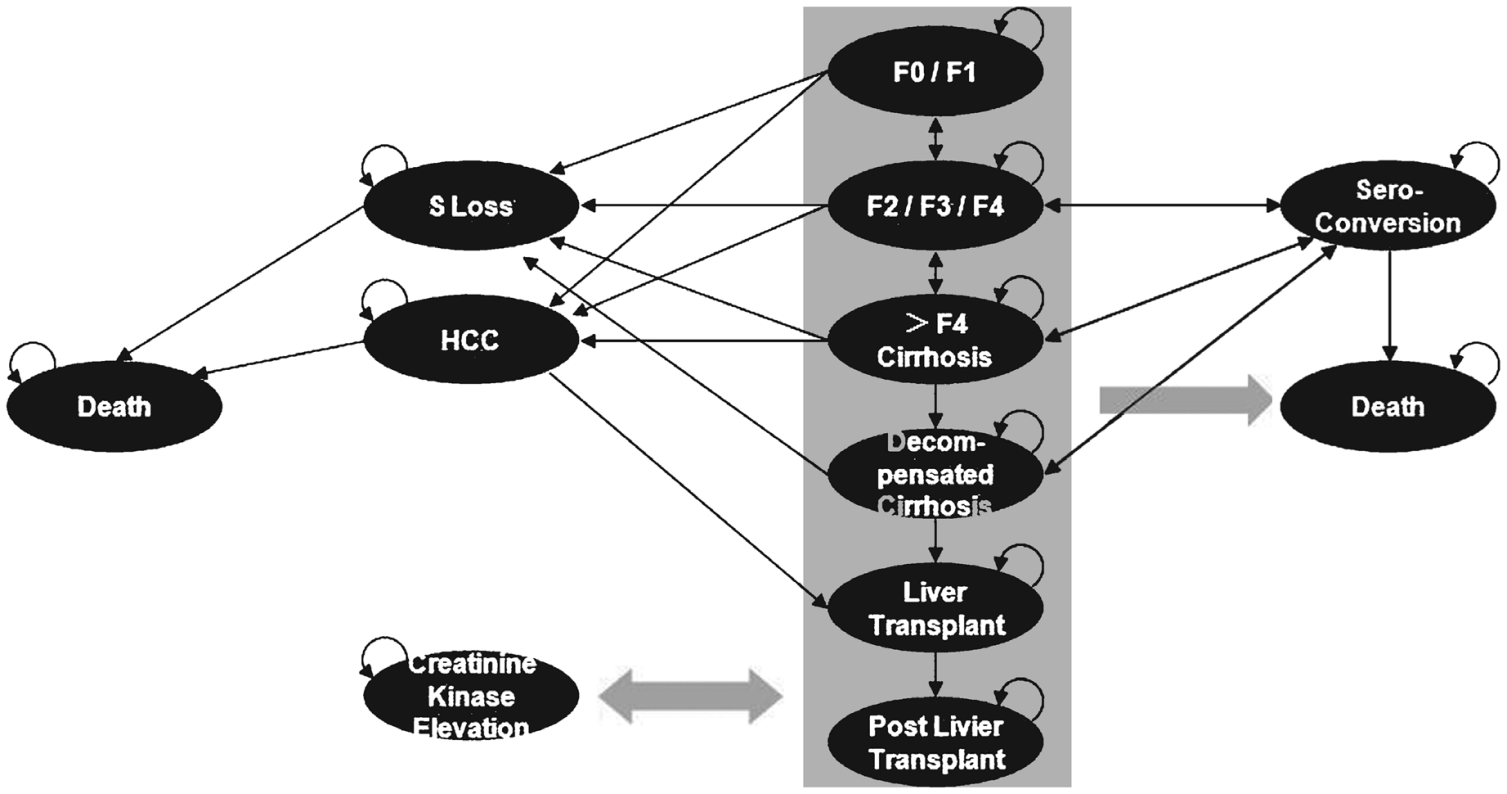
Model sub-structure. Single arrows represent transitions between health states; looped arrows indicates situations where transition out of a particular disease state is not 100% (i.e. a patient may remain in the disease state); wide arrows represent transitions to or from any state in the central green column to a specific disease/end state. Chronic hepatitis B disease states are based on Ishak fibrosis scores: F0/F1 (mild disease), F2/F3/F4 (fibrosis), >F4 (advanced fibrosis/compensated cirrhosis), decompensated cirrhosis, hepatocellular carcinoma, liver transplant, post-liver transplant. Adverse events/other dynamic disease states include HBsAg loss, HBeAg seroconversion, creatine kinase elevationThe end state is death.

The main model used four (two-by-two combination) states determined by combinations of treatment response (controlled/uncontrolled viral load) and drug resistance (yes/no). A controlled viral load state indicates HBV DNA level of <300 copies/ml in all treatment options. All patients started in model with uncontrolled viral load. Within each of the four combinations of treatment response and drug resistance, a sub-model was designed around CHB disease states based on available natural history data and end states that the patients progressed through (Figure 2). The natural progression rate through these states was dependent on viral load status, being faster with uncontrolled viral load, and used a weighted ‘uncontrolled’ viral load average disease progression based on results of the REVEAL study [8], [9]. Progression through Ishak fibrosis stages F2–F4 was modelled using the data from the analysis of Veenstra et al. and calibrated using the REVEAL study [9], [10]. REVEAL study was selected because of its long-term observation period extending over 13 years among Asian subset, while Veenstra analysis was selected because no Asian subset studies were available with such uniquely large patient number. Rates of progression to end-states were estimated using clinical trial data [10]. Mortality due to severe CHB disease states was based on the published evidence [4], [10]–[13], whereas mortality rates similar to the general population of any country were assumed for less severe CHB disease states (F0/F1, F2/F3/F4, HBeAg seroconversion, HBsAg loss).

### Time horizon

Events and costs were modeled over a time horizon of 30 years in yearly cycles, with the base case treatment duration of 5 years. The 5-year treatment includes all treatment costs associated with salvage therapy for patients who have developed resistance and/or adverse events. Patients reaching treatment endpoints such as HBeAg seroconversion do not incur treatment cost during this period. For the 25-year calculation, only management costs associated with disease progression are incorporated.

PVA analysis relies on the availability and extent of solid clinical data. However, most clinical data are available for 5 or 6 years at most, not as long as the 30 years of simulation period. This is a key reason for the 5-year treatment duration and 25-year follow-up time horizon of the analysis. For the purpose of simulation across all comparisons, we assumed that the 5-year end-state patient clinical status is preserved at sustained viral load suppression and resistance until year 30.

### Patient population

Patient population at baseline was set to most closely reflect the average Korean population. Utilizing the data available, the hypothesized average values were validated through an expert panel and tested through sensitivity analyses. The patient population is comprised of cohorts of 1,000 hypothetical hepatitis B e antigen (HBeAg)-positive and HBeAg-negative, CHB patients for each treatment group. The ratio of HBeAg-positive to negative patients for model results is 60% to 40% (Adapted from Kim et al. 2010 KASL, originally 56%:44%) [2]. The percentage of those entering the model in ‘cirrhosis’ state was assumed to be 17% [2]. All other individuals are assumed to start in ‘F2/F3/F4’ state. Patients with an Ishak fibrosis score F0/F1 were assumed not to initiate treatment (APASL treatment guidelines and guidance from key opinion leaders)

Analysis of disease progression was separately run for the HBeAg-positive and negative CHB patient population. Results could be produced for either population, with the model defaulting to an average of the two populations. Average age of CHB treatment patients was assumed to be 35 years at treatment initiation, regardless of HBeAg status.

### Clinical & mortality input

Viral load suppression data were taken from pivotal trials for lamivudine [14]–[19], telbivudine [19]–[21], entecavir [17], [18], [22], [23]. For switch to entecavir of suboptimal responders for lamivudine, Heo et al. data was mainly utilized [24].

It was assumed that reversal of fibrosis did not occur while off-treatment or under uncontrolled viral load (HBV DNA >300 copies/ml). For the comparison with no treatment, the entecavir histological improvement rate was based on 6-year trial data of ≥1 score improvement on the Ishak scale [25]. For the comparison with other antiviral therapies, histological improvement rate was assumed that it is solely dependant on the percentage of controlled population. The histological improvement rate of each treatment was calculated based on the annual probability of entecavir and controlled population. Long-term resistance data was also taken from pivotal trials for lamivudine, telbivudine, and entecavir [26]–[28]. For suboptimal switch to entecavir, Heo et al. was utilized [24]. Study assumptions were summarised in Table 1. Mortality due to severe CHB disease state (Decompensated cirrhosis, HCC) was considered in the model based on published evidence [4], [9]–[13]. For less severe CHB disease states (fibrosis) mortality rates similar to the general population were assumed. Inputs for mortality rates can be also found in Table 1.

**Table 1:** Model Assumption.

Variables	Baseline Value, %	Reference
**Baseline characteristics**		
HBeAg-postivie: HBeAg-negative (% of population)	60%∶40%	[2]
Ishak fibrosis stage F2∼F4: >F4^a^	83%∶17%	[2]
Treatment compliance rate	74%^b^	[38]
Annual discount rate	5%^b^	HIRA 2010
**Annual disease progression rates – HBeAg-positive and –negative (unconrolled : controlled viral load)**		
Progression from F0/F1 to F2/F3/F4	0.5%^c^∶0.0%^d^	[9], [10]
Progression form F2/F3/F4 to >F4/cirrhosis	7.0%^c^∶1.9%^d^	[9], [10]
Progression from>F4/cirrhosis to decompensated cirrhosis	3.1%^c^∶0.8%^d^	[9], [10]
Progression from decompensated cirrhosis to liver transplant	2.6%^c^∶0.7%^d^	[9], [10]
Progression from liver transplant to post liver transplant	100.0%^c^∶100.0%^d^	Assumption
Progression from HCC to liver transplant	30.0%^c^∶30.0%^d^	[10]
F0/F1 to HCC	0.5%^c^∶0.1%^d^	[11]
F2/F3/F4 to HCC	1.0%^c^∶0.2%^d^	[9]
>F4/cirrhosis/advanced fibrosis to HCC	2.2%^c^∶2.2%^d^	[11]
Decompensated>F4 /cirrhosis/advanced fibrosis to HCC	2.2%^c^∶2.2%^d^	[11]
**Annual death rates**		
Year 1 to 30(Age 35 to 64)^e^	0.1–1.19%	WHO 2009
>F4/cirrhosis/advanced fibrosis to death	4.9%^e^	[12]
Decompensated>F4/cirrhosis/advanced fibrosis to death	19.0%^e^	[4]
HCC to death	23.0%^e^	[9], [10]
Liver transplant to death	13.0%^e^	[13]
Post-liver transplant to death	2.5%^e^	[13]
**Annual histological improvement rates - on treatment – HBeAg-positive and –negative (uncontrolled:controlled viral load)**		
Improvement from F2/F3/F4 to F0F1	0.0%^c^∶18.5%^d^	[25]
Improvement from >F4 /cirrhosis/advanced fibrosis to F2/F3/F4	0.0%^c^∶20.0%^d^	[25]

a No patients entered the model in Ishak fibrosis stages F0/F1 or decompensated cirrhosis/HCC/liver transplant/post-liver transplant.

b Sensitivity analysis input range for compliance rate 70%∼90%, for annual discount rate 3%∼7%, and for all other variables ±10% standard deviation.

c Patients with uncontrolled viral load (HBV DNA level>300 copies/ml).

d Patients with controlled viral load (HBV DNA level <300 copies/ml).

e Assumptions for the population as a whole. The probability of death was linked to liver histology and not to viral load.

HBeAg = hepatitis B e antigen; HBsAg = hepatitis B surface antigen, HBV = hepatitis B virus, HCC = hepatocellular carcinoma; CHB = chronic hepatitis B; HIRA = Health Insurance Review & Assessment Service in Korea

### Cost inputs

The model assumed that each disease state has associated cost of care. This study only considers direct healthcare cost such as the cost related to diagnosis of the treatment, laboratory testing, drugs, follow-up and disease complication. Drug costs were taken from the database from HIRA (Health Insurance Review & Assessment Service in Korea). Disease complication costs were updated using current clinical practice and HIRA data [29] (Table 2). The analysis was performed from the perspective of national health system. For management of resistance, the additional treatment cost was considered; including clinical assays for mutant detection as well as the salvage therapy cost.

**Table 2:** Model Cost Input.

Variables	Cost (USD)	Source
**Disease state cost** a		
F0/F1	339^b^	[29], Updated based on Yang et al. 2010 with HIRA 2009 data
F2/F3/F4	373^b^	[29], Updated based on Yang et al. 2010 with HIRA 2009 data
>F4 advanced fibrosis/compenstated cirrhosis	702^c^	[29], Updated based on Yang et al. 2010 with HIRA 2009 data
Decompensated cirrhosis	1,474 c	[29], Updated based on Yang et al. 2010 with HIRA 2009 data
Liver transplantation	78,684^d^	KONOS (Korean Network for Organ Sharing) database
Post liver transplantation	11,697 d	KONOS (Korean Network for Organ Sharing) database
HBsAg loss	170^e^	[29], Updated based on Yang et al. 2010 with HIRA 2009 data
Hepatocellular carcinoma	5,224	[29], Updated based on Yang et al. 2010 with HIRA 2009 data
Death	0^f^	-
**Antiviral treatment cost per pill**		
Entecavir	5.88	HIRA 2010
Lamivudine	3.26	HIRA 2010
Telbivudine	3.35	HIRA 2010

a Antiviral drug cost not included

b Calculated from fibrosis total cost, assuming F0F1/∶F2F3F4 = 1∶1.1 based on guidance of Korean advisory board, In addition, patient ratio is assumed to 1∶1

c Calculated from cirrhosis total cost, assuming compensated∶ decompensated  = 1∶2.1 and patient ratio is assumed to be 1∶1.61 based on Yang et al. 2004

d Calculated & updated liver transplant surgery cost & post liver transplant cost with Korea organ sharing networks database & Seoul national university organ transfer center data base

e Assume about 50% of F0/F1 based on EASL guideline

f Only consider direct medical cost of caring disease state

For drug-related adverse events, the model assumed that safety and tolerability of entecavir were similar to those of lamivudine and the drug has no requirement for renal monitoring (Entecavir SPC 2009). However, patients receiving telbivudine may be at the risk of the elevation of creatine kinase concentration that requires additional monitoring and treatment. Specifically telbivudine requires additional creatinine clearance/serum phosphate test during the treatment period [30], [31].

Costs were calculated to net present value at an annual discount rate of 5%, according to HIRA guideline recommendations. All costs were inflated to 2010 prices using Korea health-specific consumer-prices indices. All costs were calculated from the holistic point of view, with both patients’ out-of-pocket cost and payor's cost in consideration.

### Model outputs

The primary outcome measures were per-patient cost per day of treatment of entecavir compared with no treatment or other antiviral therapies over a time horizon of 30 years assuming 5-year duration of treatment. The daily overall treatment cost was based on drug acquisition cost and clinical differences between treatment options. In comparison with no treatment, clinical events considered were HBsAg loss, HBeAg seroconversion, fibrosis, cirrhosis, decompensated cirrhosis, HCC, liver transplantation and post-liver transplantation. In comparison with other antiviral therapies, clinical attributes considered were long term slowing of disease progression and reversal of liver fibrosis/cirrhosis, high genetic barrier and long-term resistance, for which additional monitoring and pharmacotherapy cost, and related adverse events cost are avoidable.

Costs avoided for each of the clinical events listed above over the 30 year follow-up period were calculated for use of entecavir compared with no treatment or other antiviral therapies and then re-calculated to estimate the ‘cost saving per day of treatment’ (cost saving/total days on treatment). Costs avoided were calculated for each event type avoided as (total number of events avoided×cost of event/days on treatment). The resultant costs were applied to the daily dose acquisition cost for each treatment to determine the true cost of entecavir relative to no treatment or other antiviral therapies for which the model was run. In this manuscript, costs are presented in 2010 values and as the proportion of entecavir acquisition cost saved by use of entecavir relative to the comparators. In this analysis, we assume that 1 USD is equal to 1,000 KRW.

### Sensitivity analyses

Both univariate and multivariate analyses were conducted to test the robustness of model outcome, given the uncertainty around certain clinical and cost inputs. The univariate analysis investigated the effects of varying one key input at a time and then ranked the influence of each variable, and the multivariate analysis investigated the effects of varying all variable inputs simultaneously. The analyses results were used to identify the key inputs generating the biggest variation in model outputs. These inputs, along with other key inputs with some uncertainty, were subsequently used for probabilistic sensitivity analyses. Discount rate, all unit costs and disease state costs, clinical profiles including virologic suppression efficacy and resistance, baseline characteristics such as HBeAg positive to negative ratio, and compliance rate were among the key input variables that were tested.

## Results

### Entecavir vs. No Treatment

**Figure 3:**
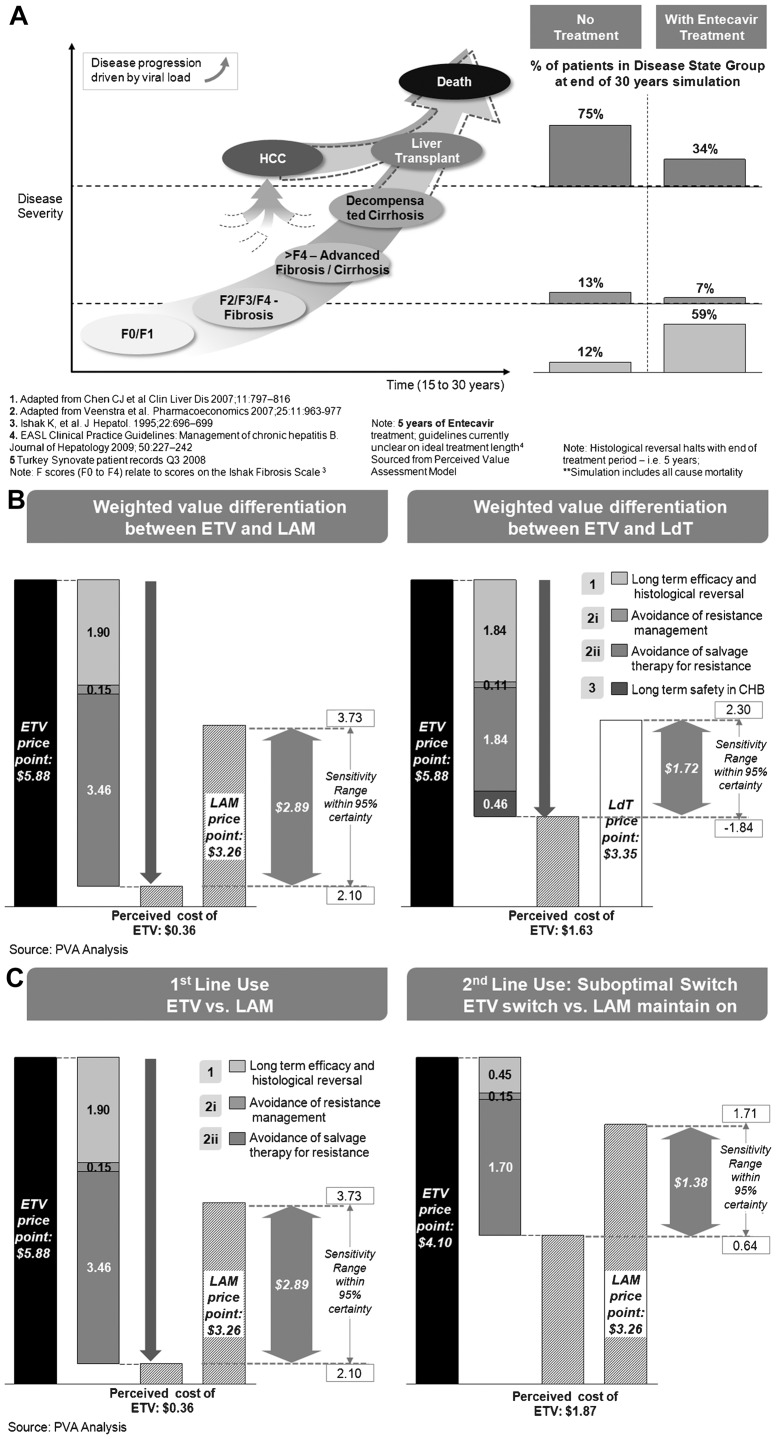
Model Output. (A) Simulation of No treatment vs. treatment with entecavir for a Korean CHB population: Model simulation of patient outcomes at year 30 in two hypothetical Korean cohorts of patients with chronic hepatitis B, 60% of whom were HBeAg-positive. A total of 1000 patients were untreated and 1000 patients received entecavir treatment for 5 years (B) Weighted value differentiations between entecavir versus lamivudine or telbivudine: Daily cost savings per patient with chronic hepatitis B (60% with HBeAg-positive CHB) in Korea over a 30-year period by use of entecavir instead of lamivudine or telbivudine assuming an average patient lifespan of 65 years and an average initiation age of 35 years and 5 years of treatment with 74% compliance (histological reversal stops when treatment stops). Sensitivity analysis was conducted within 95% Confidence Interval. (C) Weighted value differentiations between switching to entecavir versus maintaining on lamivudine of suboptimal responders for lamivudine: Daily cost savings per patient with chronic hepatitis B (60% with HBeAg-positive CHB) in Korea over a 30-year period by use of entecavir instead of maintain on lamivudine assuming an average patient lifespan of 65 years and an average initiation age of 35 years and 5 years of treatment with 74% compliance (histological reversal stops when treatment stops). Sensitivity analysis was conducted within 95% Confidence Interval.

If CHB is untreated, the disease can progress to serious complications such as decompensated cirrhosis, HCC, and even death. Optimal antiviral treatment can slow down, or even reverse, the disease progression through effective and sustained virologic suppression, long-term protection against resistance to treatment, and long-term safety with low incidence of associated adverse events. Entecavir exhibits the optimal profile in all clinical aspects among CHB antiviral NA therapies available in Korea. This observation coincides with the patient distribution comparisons after the modelling period. Simulation of disease progression over 30 years showed that, when patients were not treated, death, liver transplantation, or HCC occurred in 75% of the patients by year 30; advanced fibrosis/cirrhosis or decompensated cirrhosis developed in 13%; and 12% remained in stages F0 to F4 fibrosis (Figure 3A). On the other hand, for entecavir treated patients, 34%, 7% and 59% of the patients ended in serious, medium and mild stages, respectively (Figure 3A). In other words, treatment with high genetic barrier antiviral therapy significantly reduced disease progression in CHB natural history. Saving in CHB management-related costs over the patients' lifetime achieved by using entecavir was $8.98, which fully outweighed the acquisition cost of the drug ($5.88). This indicates the use of entecavir instead of no treatment achieved daily saving of $3.10 (95% confidence interval∶ $2.10∼$3.76).

### Entecavir vs. Lamivudine or Telbivudine

When costs relating to long-term virological suppression rate, treatment of resistance, and drug-related toxicity for entecavir relative to lamivudine or telbivudine were taken into consideration, the difference in acquisition cost was more than offset by costs avoided with the use of entecavir (Figure 3B). Daily acquisition cost of lamivudine and telbivudine is $3.26 and $ 3.35, respectively. When considering the clinical benefit of entecavir, such as long-term virological suppression and high genetic resistance, the use of entecavir represented daily cost saving of $2.89 (95% confidence interval: $2.10∼$3.73). In the case of comparison between entecavir and telbivudine, the daily cost saving of entecavir compared to telbivudine was $1.72 (95% confidence interval: $−1.84∼$2.30) due to superior clinical profile of entecavir in terms of virological suppression rate, high genetic barrier, and long-term safety.

### Switch to entecavir vs. maintain on lamivudine for suboptimal responders for lamivudine

In this analysis, 1 year lamivudine use was included into model treatment duration, and then it was assumed that only suboptimal responders who did not develop lamivudine resistance among total population, (38.4% of total simulation population, which is weighed-averaged of 60% HBeAg positive and 40% HBeAg-negative population) were switched to entecavir or maintained on lamivudine. For 30-year simulation period, the switch to entecavir group represented daily $1.38 (95% confidence interval: $0.64∼$1.71) cost saving compared to the maintained-on lamivudine (Figure 3C).

### Sensitivity analysis

Sensitivity analyses were conducted to assess the result robustness across different variables. Multivariate sensitivity analysis as well as univariate sensitivity analysis indicated that the annual number of pills taken (dosage compliance rate) was the most sensitive variable, above discount rate, clinical inputs, cost inputs, or any other inputs. Entecavir remained cost saving despite a±10% variance in pill count (in comparison with no treatment, daily cost saving were $3.76 when pill count was reduced by 10% and $2.10 when pill count went up by 10% from the default value of 270 pills per year).

## Discussion

The largest number of Korean patients infected with HBV was exposed to major health risks and high financial burdens, not to mention the subsequent development of cirrhosis and HCC [32], [33]. Lamivudine, telbivudine, and entecavir are the three major oral agents currently used in Korea for the treatment of chronic HBV infection. As these treatment options often require long-term use, the cost of these options should be taken into account when formulating recommendations. There are several guidelines regarding various options for CHB treatment, but they do not consider the cost-effectiveness/benefit of these therapies. Therefore, we evaluated the cost benefit of entecavir, the agent known for its superior clinical profile, compared with the other available antiviral agents for the treatment of CHB based on a best available clinical trial data. As tenofovir is not yet available in Korea, it was excluded from analysis. The results of our analysis suggest that initiating therapy with high genetic barrier such as entecavir improves health outcomes in a cost-benefit manner compared with no treatment or other low genetic barrier antiviral agents. Our findings were driven by several clinical differentiation factors: (i) greater viral suppression level, (ii) the higher genetic barrier in resistance, (iii) the long-term safety. The PVA model demonstrates how these clinical outcomes translate into economic value. The PVA model, therefore, allows the economic differentiation based on the identified ‘value drivers’.

In this analysis, considering both the cost of treatment and the cost saved as a result of treatment with entecavir relative to either no treatment or treatment with other antiviral agents, entecavir was shown to be most cost saving in Korea. This is mainly because of a reduction in the incidence of, and therefore potential costs incurred by, late-stage liver disease in comparison with comparators.

The simulation of treating with entecavir versus no treatment predicted improved clinical outcomes for entecavir-treatment patients over a 30-year time period. Progression to HCC, liver transplant or death was estimated at 75% for patients without treatment compared to 34% for patients receiving entecavir; regression to mild disease states (F0/F1) or fibrosis (F2-F4) was 12% and 59% for these populations, respectively. The cost of not treating patients with CHB was estimated at $8.98 per day (average over patient life time). Entecavir treatment was translated into specific patient benefit in terms of events avoided, with an estimated cost saving of $3.10 per day of entecavir treatment. In Korea, CHB is particularly prevalent with approximately 1.4 million patients chronically infected (approximately 3% of total Korean population). Among them, it is reported that only 10.7% of patients diagnosed as CHB were appropriately monitored or treated (Synovate Market Research Korean Study – HBV monitor and treatment pattern). CHB infection is a significant cause of morbidity and mortality which is associated substantial amount of caring cost. Particularly, 70% of liver cancer was caused from CHB in Korea (Korea ministry of health & welfare 2010). Our analysis shows that if all untreated patients were assumed to be treated, approximately $ 1.4 billion of healthcare budget saving could be expected annually.

According to the recommendation by the Korean Association for the Study of the Liver, entecavir is preferred to lamivudine and telbivudine as a naive patient treatment due to its potent viral suppression and favourable resistance profile. However, entecavir is one of the most expensive agents in Korea (entecavir $ 5.88, lamivudine $3.26, telbivudine $3.35). Nonetheless, our economic evaluation demonstrates that naive treatment use of entecavir is the most cost-benefit strategy for managing CHB with nucleosides. The daily cost savings of using entecavir versus lamivudine and telbivudine were estimated at $ 2.89 and $ 1.72, respectively, even considering antiviral acquisition cost.

In our analysis, we also investigated the cost-benefit of switch to entecavir for lamivudine pre-treated patients. According to the latest guideline of the Korean Association for the Study of the Liver, in the case of suboptimal early virological response to antiviral agent with low genetic barrier, either a switch to another antiviral agents with higher genetic barrier of resistance or add-on another antiviral agents with no cross resistance developed is the recommended treatment of choice. Lamivudine has been known for its high non-responding rate and resistance rate with 64% and 23%, respectively, in HBeAg-positive patients for 1 year treatment [17], [19]. There are several clinical trials regarding this switch. [34] The recent study of Heo et al. assessed the efficacy and resistance rate of switching Korean suboptimal responders from lamivudine monotherapy to entecavir 1 mg/day [24]. In this analysis, the switch group showed 61.1% undetectable level of HBV DNA, while maintain-on group remained 11.4% of undetectable level within a year. The resistance rate of switch group and maintain-on group were 0% and 34.4%, respectively, within the same time period. Based on this clinical trial result, we investigated the economic value of suboptimal switch. Even considering the difference in acquisition cost, the switch group showed daily cost saving of $1.38 compared to maintain-on group. This cost saving ($1.38) was lower than the one in naive patient treatment ($2.89). Our finding supports the fact that earlier use of more potent drug with higher virological suppression and genetic barrier is more economically beneficial than later use.

The current standard for assessing the value of different treatment options is the cost-effectiveness analysis. Quality-adjusted life-years (QALYs) are often used in these analyses to allow comparisons between different treatments and disease states. Cost-effectiveness analyses involving treatment for CHB include the studies of Calcagno et al. [35] and Veenstra et al. [10], which showed entecavir to be cost-effective compared with lamivudine alone or with adefovir salvage or combination therapy in the US and Asia Pacific. The study of Lui et al. [36] showed that, in HBeAg-positive patients, lamivudine roadmap was most cost-effective. In HBeAg-negative patients, entecavir and tenofovir monotherapies were more cost-effective than the roadmap models. However, cost-effectiveness analyses have some limitations as the clinical benefits of a given treatment for specific patients are difficult to assess when the output is expressed in QALYs. Additionally, when thresholds for cost-effectiveness are used, the actual cost or cost averted for treating a patient with the treatment choice is unclear. Value-based approaches avoided these limitations as they compared the cost of an intervention to the cost saved as a result of that intervention relative to another course of action.

This model has been developed as an alternative to cost-effectiveness analysis to help the healthcare decision makers evaluate the ‘value for money’ of treating CHB patients with several antiviral agents available in Korea. The model is robust, as health economic approaches were leveraged to create a detailed analysis that holds up under scrutiny and it is based on best available data. Importantly, the model assumed that optimal treatment controls viral load and reverses liver fibrosis. In addition, the model was run over a time period of 30 years because of the increased risk of progression to long-term complication, including cirrhosis, decompensated liver disease and HCC in patients with CHB.

While certainly robust, the model has some limitations. The model approach neglects the cost of social burden and considers direct medical costs only. Hence, the lower death rate would undermine the cost benefits of entecavir since no disease state cost is assumed for incidence of death (Table 2). Entecavir, a therapy with a lower death rate, would apparently incur more healthcare cost and lead to lower cost benefits than one with a higher death rate. Another limitation of the model, as with the cost-effectiveness analysis or any other lifelong simulation, is that due to lack of clinical data that extends beyond 5 to 6 years, the 30 years time horizon is hypothetically based on 5-year treatment duration and 25-year follow-up duration. Cost benefit becomes more apparent under long-term observation for chronic cases such as CHB. As such, the 5-year results were extended to the entire simulation period. Such practice is common in cost-utility assessment of CHB [4], [13], [14], [37]. For the purpose of simulating the long-term economic effect of the 5-year treatment, we assumed that the 5-year patient end-state virologic status is maintained until year 30 instead of assuming termination of treatment. Thus, viral load rebound, seroconversion rebound, and other clinical events associated to treatment termination were disregarded.

In conclusion, economic benefit of each CHB treatment option has been analyzed by novel cost estimation model. Based upon this analysis, high genetic barrier antiviral such as entecavir exhibited the highest cost saving compared to alternative treatment strategies mainly due to its superior clinical profile, even considering expensive price point.
